# Dichloroacetate is an effective treatment for sarcoma models *in vitro* and *in vivo*

**DOI:** 10.1186/2049-3002-2-S1-P9

**Published:** 2014-05-28

**Authors:** Melissa Rooke, Lucy A Coupland, Thy Truong, Anneke C Blackburn

**Affiliations:** 1 John Curtin School of Medical Research, Australian National University, Canberra, ACT, Australia; 2 Mass Spectrometry Facility, Australian National University, Canberra, ACT, Australia

## Background

Sarcomas are cancers that arise from tissues of mesenchymal origin and there has been limited improvement in treatments over the last 30 years. The Warburg effect is a widespread metabolic phenotype of cancer, where glycolysis is favoured despite the presence of oxygen. Dichloroacetate (DCA) is a pyruvate dehydrogenase kinase (PDK) inhibitor in clinical use that can reverse the Warburg effect, inhibiting growth and enhancing apoptosis in a range of cancers. We have investigated its effectiveness against sarcoma cells *in vitro* and *in vivo*.

## Materials and methods

Three cell lines (mouse fibrosarcoma S180, mouse osteosarcoma K7M2 and human fibrosarcoma HT1080-luc2) were examined for cell viability after DCA treatment *in vitro* (neutral red uptake assay), alone and in combination with doxorubicin. *In vivo*, K7M2 cells were injected s.c. into Rag1^-/-^ mice (2 sites per mouse, 7-9 mice per group) and established tumours were treated with DCA in the drinking water (0, 0.5, 1.0 and 1.5 g/L, delivering 0, 70, 125 and 165 mg/kg/day, respectively). Tumour growth was monitored with callipers. Plasma was collected on d1 and d15 for measurement of DCA levels (LC-MS).

## Results

DCA significantly reduced the total viable cell number after 48 h of treatment in the mouse sarcoma lines (~15% at 0.5 mM DCA, and 30-40% at 5 mM DCA), however HT1080-luc2 cells showed only a 10% reduction in cell number with 5 mM DCA. There was no morphological indication of apoptosis, suggesting DCA was decreasing proliferation. Chronic treatment of the mouse cells (5 mM DCA for 2 weeks) resulted in significantly slower growth rates as measured over 48 h (7 and 13% total cell number compared to untreated S180 and K7M2 cells, respectively). DCA did not synergise with doxorubicin but was additive at lower concentrations of doxorubicin. *In vivo*, 1.0 and 1.5 g/L DCA significantly reduced tumour growth (33.0 and 33.1% reduction in tumour size on d13, p=0.001 and 0.01 respectively). Plasma DCA was undetectable on d1 of treatment, but by d15, 1.0 and 1.5 g/L DCA delivered 2-44 uM. Tumour DCA concentrations were also measured and found to be in the range of 25-470 uM, much lower than those typically used in *in vitro* studies.

## Conclusions

DCA was effective against an *in vivo* sarcoma model, with tumour DCA concentrations in the micromolar range. These concentrations are achievable clinically, thus DCA warrants further investigation for sarcoma treatment.

